# COPD, stage and treatment in a large outpatient clinic

**DOI:** 10.1080/20018525.2017.1267470

**Published:** 2017-01-04

**Authors:** Claire Præst Holm, Jakob Holm, Annette Nørgaard, Nina Godtfredsen

**Affiliations:** ^a^ Department of Respiratory Medicine, Copenhagen University Hospital, Hvidovre, Denmark; ^b^ Department of Endocrinology, Copenhagen University Hospital, Hvidovre, Denmark; ^c^ Lunge- og allergiklinikken, Hellerup, Denmark

**Keywords:** COPD, exacerbations, roflumilast, phosphodiesterase-4 inhibitor, GOLD groups, outpatients, frequent exacerbator

## Abstract

Some COPD patients suffer from frequent exacerbations despite triple inhalation treatment. These frequent exacerbators should be identified, as exacerbations often lead to decreasing lung function and increasing mortality. Roflumilast reduces exacerbations in patients with a previous history of exacerbations. Our aim was to describe COPD patient characteristics and compare roflumilast treatment eligible to non-eligible patients. An observational cross-section study was conducted. Patients were included from a large COPD outpatient clinic. Information regarding COPD patient characteristics was registered on a standardized form and lung function was measured. Patients were categorized according to the GOLD classification. Eligibility for roflumilast treatment was assessed and patient characteristics compared between groups. 547 patients were included. Most patients (54%) were in GOLD group D. 62 patients (11.3%) met the criteria for treatment with roflumilast. Among the patients eligible for roflumilast treatment, only 14 patients (22.6%) were receiving treatment. There were no significant differences in FEV_1_, number of exacerbations, hospitalization due to exacerbation, MRC grade, age, smoking status and medication use between patients receiving roflumilast and not treated eligible patients. Our study documents low use of roflumilast treatment. In view of the established effect of roflumilast we think that this treatment should be considered more consistently as an option among COPD patients fulfilling the criteria for this therapy.

## Introduction

Some chronic obstructive pulmonary disease (COPD) patients suffer from frequent exacerbations despite triple-treatment with inhaled corticosteroids (ICS), long-acting β2-agonists (LABA) and long-acting muscarinic antagonists (LAMA). As a history of previous exacerbations [1] has been found to be the best predictor of future exacerbations, early identification of these patients is important in order to optimize individual treatment. Recently, phenotypical categorization of COPD patients based on clinical features has become more widespread. This categorization might have the possibility of enabling more individualized, pharmacological treatment. The Evaluation of COPD Longitudinally to Identify Predictive Surrogate Endpoints (ECLIPSE) study identified different COPD phenotypes including the ‘frequent exacerbator’.[1] Exacerbations are known to have a detrimental effect on patient-related outcomes, often leading to decreasing lung function [2] and increasing mortality.[3] Furthermore, patients with frequent exacerbations are found to have a reduced response to therapy and a higher level of inflammatory serum markers as compared to other phenotypes.[4] This persistent inflammation has been proposed as an explanation of the reduced lung function observed in these patients.[4]

Roflumilast is an oral phosphodiesterase-4 (PDE-4) inhibitor that reduces inflammation [5] and reduces exacerbations in patients with COPD. It is approved for the treatment of COPD in patients with forced expiratory volume in 1 s (FEV_1_) of ≤ 50% predicted, chronic bronchitis and a history of at least two exacerbations in the previous year.[6] The expenses for the drug can only be reimbursed if the patients fulfil all of the above criteria.

The present study was conducted in one of Denmark’s largest COPD outpatient clinics, Copenhagen University Hospital, Hvidovre. Our aim was to describe patient characteristics in terms of lung function, presence of chronic bronchitis, treatment and exacerbations in a large cohort of COPD-patients. Prevalence of COPD according to The Global Initiative for Chronic Obstructive Lung Disease (GOLD) groups A–D was assessed. Furthermore, prevalence of treatment with roflumilast in patients eligible for this medication was investigated.

## Methods

**Figure 1.**
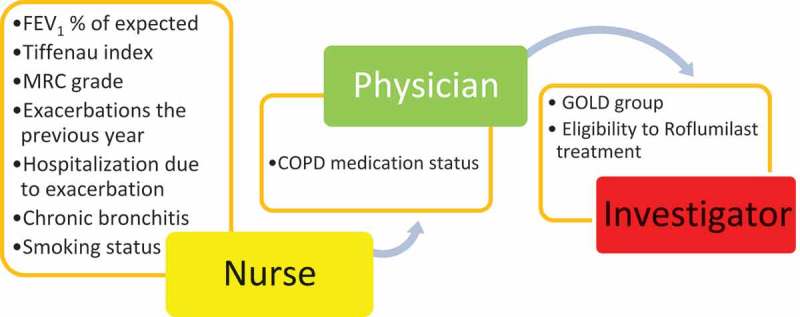
Summary of collection of data. Abbreviations: FEV_1_, forced expiratory volume; MRC-grade, Medical Research Council questionnaire; GOLD, the Global Initiative for Chronic Obstructive Lung Disease.

The study was an observational cross-section study. Patients were included in a six-month period from 1 January to 30 June 2015. All patients with a diagnosis of COPD seen by a physician in the outpatient clinic situated at the Copenhagen University Hospital, Hvidovre were eligible to participate in the study. If patients had more than one visit during the inclusion period only results from the last visit were included in the analysis. There were no further inclusion or exclusion criteria. Information regarding lung function, degree of dyspnea, smoking habits, exacerbations the previous year and hospitalization for these, and presence of chronic bronchitis was collected and registered on a standardized form (Figure 1). COPD specific medication status and possible change in medical treatment at the current visit was registered. Patients were subsequently categorized according to the GOLD groups A–D and determination of eligibility for treatment with roflumilast was registered.

The degree of dyspnea was determined according to the Medical Research Council Questionnaire (MRC). Chronic bronchitis was defined as coughing and sputum on most days for ≥ 3 consecutive months for at least two consecutive years.[7] Exacerbations were registered as 0, 1, 2, 3 or ≥ 4 the previous year and defined as episodes of worsening requiring treatment with prednisolone and/or antibiotics or hospital admission. Use of COPD related medication was recorded as current use of short-acting β_2_-agonists (SABA)/short-acting muscarinic antagonists (SAMA), LAMA, LABA, ICS, prednisolone and/or antibiotics for acute exacerbation or other treatment. Finally, former and present use of roflumilast was registered. Prescription of roflumilast is exclusively performed by respiratory specialists (or after conferral with such) and are in accordance with guidelines from the Danish Medicines Agency. Spirometry was performed using Spirotrac® manufactured by Spiropharma. The spirometers were calibrated every morning.

Statistical analysis was conducted using SAS version 9.4. Baseline characteristics are presented as numbers and percentages of observations for categorical variables. Continuous variables are presented with means or medians with corresponding standard deviations or inter quartile range. Comparisons between groups were performed using unpaired two-sample t-test, Wilcoxon two sample t-test, Fisher’s exact test or χ^2^-test as appropriate. In case of unequal variances, unequal variances t-test was used instead of the regular t-test. Significance was defined as a two-sided *p*-value ˂ 0.05.

The project was approved by the relevant Danish authorities including the Danish data protection agency.

All patients provided written informed consent before inclusion in the study.

## Results

During the six-month inclusion period 580 forms with written consent were collected. Of those, 33 had filled forms on two separate occasions. This led to 547 patients being included in the study.

**Figure 2.**
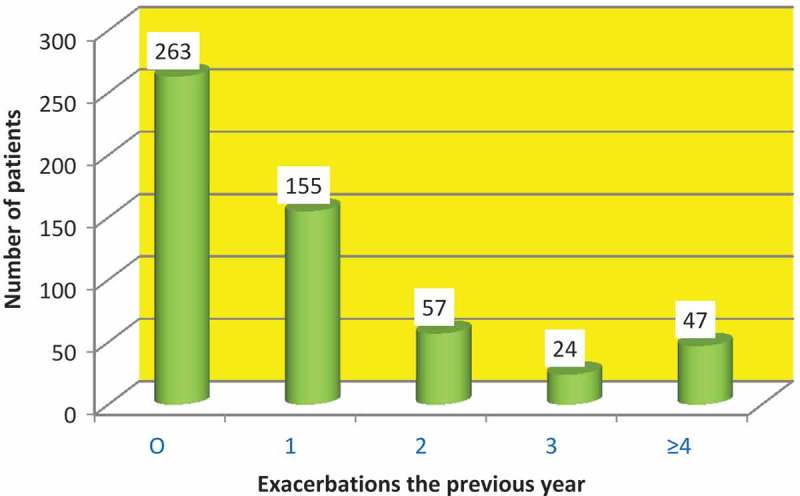
Distribution of the number of exacerbations in the previous year.

Most patients (*n* = 296) were in GOLD group D, but there was also a large proportion (*n* = 95) in GOLD group A (Table 1). The mean prevalence of exacerbations was 1, and 28% of the patients had been hospitalized due to an exacerbation. The distribution according to the number of exacerbations is shown in Figure 2.

**Table 1.** Patient charecteristics.

	All patients (*n* = 547)	Patients not eligible for roflumilast (*n* = 484)	Patients eligible for roflumilast (*n* = 62)
Age, mean (SD)	68.9 (±10.5)	69.0 (±10.5)	67.9 (±9.9)
FEV_1_ % predicted, mean (SD)	45.5 (±16.3)	47.0 (±16.3)	33.7 (±10.7)
Female sex, *n* (%)	311 (57.0%)	275 (56.9%)	36 (58.1%)
MRC-grade, median (IQR)	3 (2;4)	3 (2;4)	4 (3;5)
Number of exacerbations, median (IQR)	1 (0;1)	0 (0;1)	3 (2;4)
Hospitalization due to exacerbation, *n* (%)	152 (27,8%)	117 (24,2%)	34 (54,8%)
Chronic bronchitis, *n* (%)	274 (50.6%)	212 (44.2%)	62 (100%)
Smokers, *n* (%)	138 (25.3%)	126 (26.1%)	12 (19.4%)
GOLD group A, *n* (%)	95 (17.4%)	95 (19.6%)	0
GOLD group B, *n* (%)	57 (10.4%)	57 (11.8%)	0
GOLD group C, *n* (%)	99 (18.1%)	92 (19%)	6 (9.7%)
GOLD group D, *n* (%)	296 (54.1%)	240 (49.6%)	56 (90.3%)
SABA/SAMA, *n* (%)	457 (83.6%)	398 (82.2%)	58 (93.6%)
LAMA, *n* (%)	454 (83%)	395 (81.6%)	58 (93.6%)
LABA, *n* (%)	482 (88.1%)	421 (87%)	60 (96.8%)
ICS, *n* (%)	378 (69.1%)	319 (65.9%)	58 (93.6%)
Roflumilast, *n* (%)	14 (2.6%)	0	14 (22.6%)
Previous roflumilast, *n* (%)	11 (2%)	4 (0.8%)	7 (11.3%)
Azithromycin, *n* (%)	7 (1.3%)	4 (0.8%)	3 (4.8%)
Antibiotics for acute exacerbation, *n* (%)	25 (4.6%)	18 (3.7%)	7 (11.3%)
Prednisolone for acute exacerbation, *n* (%)	27 (4.9%)	19 (3.9%)	8 (12.9%)

SD, standard deviation; IQR, inter quartile range; FEV_1_, forced expiratory volume; MRC-grade, MEDICAL RESEARCH COUNCIL questionnaire; GOLD, the Global Initiative for Chronic Obstructive Lung Disease; SABA/SAMA, short-acting β_2_-agonists/short-acting muscarinic antagonists; LAMA, long-acting muscarinic antagonists; LABA, long-acting β_2_-agonists; ICS, inhaled corticosteroids.

Mean FEV_1_ was 45.5% and the prevalence of chronic bronchitis was 50%. More than 80% of the patients received inhalation therapy with LABA, LAMA and SABA/SAMA, and ICS was taken by 69%. Of other medication azithromycin was recorded in 1.3% of the total population (Table 1).

Sixty-two patients (11.3%) met the criteria for treatment with roflumilast. These patients had a significantly lower lung function (mean FEV_1_ was 13.3% lower in the ‘eligible for roflumilast group’ (*p* ˂ 0.001)). The MRC grade was significantly higher (*p* ˂ 0.001) and, as expected, exacerbations were more frequent (*p* ˂ 0.001) in the patients eligible for roflumilast treatment. A higher percentage of these patients had been hospitalized for an exacerbation (54.8% against 24.2%, *p* ˂ 0.001). There was no significant difference in gender (*p* = 0.87), proportion of smokers (*p* = 0.25) or in age (*p* = 0.44) between groups.

Among the patients eligible for roflumilast treatment, only 14 patients (22.6%) were receiving the treatment. One patient had received the medication previously but stopped due to side effects. Eight patients had not taken roflumilast earlier but had it prescribed during the present visit. All patients taking roflumilast were already prescribed triple inhalation therapy. There were no significant differences between the patients receiving roflumilast and those who did not (but were eligible to the therapy). FEV_1_, number of exacerbations, hospitalization due to exacerbation, MRC grade, age, smoking status and medication use were the same in the two groups.

## Discussion

We found that 54% of our cohort of patients seen at a specialist COPD outpatient clinic were in GOLD group D. We furthermore identified 11% of patients as eligible for roflumilast treatment. The proportion of eligible patients who actually received treatment was low at 22,6%.

Surprisingly many patients were in GOLD group A. A possible explanation for this could be that some of these patients have asthma-COPD overlap syndrome (ACOS) and are primarily seen at the clinic because of their asthma. GOLD group A patients are supposed to be followed by their general practitioner.

It was our experience that very few patients did not want to participate in the study. In spite of this some patients were not included. Only patients with established COPD were included in the study. This meant that some patients who had not yet been formerly diagnosed with COPD (e.g. a diagnosis was made during this particular visit), were not included in the study. Some forms (approximately 25) had missing written consent although oral consent had been given and were thus not included in the study.

The REACT study showed that the number of severe exacerbations, i.e. exacerbations leading to hospitalization or death, were reduced by 24% in the roflumilast group.[6] This effect was even more pronounced in patients who had at least one hospitalization due to exacerbation in the previous year. In that case, severe exacerbations were reduced by 35%.[8] Another study found that the re-hospitalization rate in a 30-day period following hospitalization for exacerbation was lower in the roflumilast group.[9] More than half of our patients eligible for roflumilast treatment had been hospitalized due to exacerbation the previous year. It is well established that hospitalization, especially re-hospitalization, increases mortality in COPD patients.[3] Wedzicha et al. [10] have shown that roflumilast can shift patients from the frequent exacerbator phenotype to the more stable infrequent exacerbator phenotype.

We established that 62 (11%) of our patients were eligible for roflumilast treatment, and only 22.6% of these 62 actually received the treatment. To our knowledge, the prevalence of actual treatment of roflumilast eligible candidates has not been investigated before. A possible explanation of the observed low prevalence of roflumilast treatment in our study could be that some physicians prefer azithromycin to roflumilast for the frequent exacerbators. However, since azithromycin was only used in three of these patients (4.8%) this was not the case. Another explanation could be that the patients had previously been treated with roflumilast and had terminated the treatment due to side effects. However, we did not find evidence of this, as this was only the case for a single patient. This was quite surprising in light of the prevalence of documented side effects in other studies.[6] Another possibility could be that individual physicians were reluctant to prescribe roflumilast due to personal perceived or actual experience with side effects of treatment in other patients. The rate of side effects has shown to be even higher in a real-life populations than in the pharmaceutical phase III trials studies.[11] It is possible that knowledge hereof has refrained some physicians from prescribing roflumilast to eligible patients. We did not include registration of body mass index (BMI) in our study, which might have influenced the physicians’ choice of roflumilast, as weight loss has been shown to be a common side effect.[6]

We found no differences in FEV_1_, number of exacerbations or use of medication between patients who were on roflumilast and those who were not (but were eligible to the therapy). This suggests that initiation of treatment was random and that, surprisingly, it was not the patients with the most frequent exacerbations or the lowest FEV_1_ who were started on roflumilast.

## Conclusion

Our study among 547 COPD patients from an outpatient specialist clinic showed that only 54% of the patients were in GOLD group D. Of the patient population 11% was eligible for roflumilast treatment but only 22.6% of these actually received the treatment. The number of exacerbations and the FEV_1_ did not predict prescription of roflumilast.

In view of our results documenting low use and the established effect of roflumilast treatment, we think that treatment with roflumilast should be considered more consistently as an option among COPD patients fulfilling the criteria for this therapy.

## References

[CIT0001] Hurst JR, Vestbo J, Anzueto A (2010). Susceptibility to exacerbation in chronic obstructive pulmonary disease. N Engl J Med.

[CIT0002] Donaldson GC. (2002). Relationship between exacerbation frequency and lung function decline in chronic obstructive pulmonary disease. Thorax.

[CIT0003] Soler-Cataluña JJ, Martínez-García MA, Román Sánchez P (2005). Severe acute exacerbations and mortality in patients with chronic obstructive pulmonary disease. Thorax.

[CIT0004] Perera WR, Hurst JR, Wilkinson TMA (2007). Inflammatory changes, recovery and recurrence at COPD exacerbation. Eur Respir J Off J Eur Soc Clin Respir Physiol.

[CIT0005] Beghè B, Rabe KF, Fabbri LM (2013). Phosphodiesterase-4 inhibitor therapy for lung diseases. Am J Respir Crit Care Med.

[CIT0006] Martinez FJ, Calverley PMA, Goehring U-M (2015). Effect of roflumilast on exacerbations in patients with severe chronic obstructive pulmonary disease uncontrolled by combination therapy (REACT): a multicentre randomised controlled trial. Lancet.

[CIT0007] Kim V, Han MK, Vance GB (2011). The chronic bronchitic phenotype of COPD: an analysis of the COPDGene study. Chest.

[CIT0008] Calverley PMA, Fabbri LM, Goehring U-M (2015). Effect of Roflumilast on exacerbations in patients with severe COPD and a prior history of hospitalization taking combination therapy.

[CIT0009] Fu AZ, Sun SX, Huang X (2015). Lower 30-day readmission rates with roflumilast treatment among patients hospitalized for chronic obstructive pulmonary disease. Int J Chron Obstruct Pulmon Dis.

[CIT0010] Wedzicha JA, Rabe KF, Martinez FJ (2013). Efficacy of Roflumilast in the COPD frequent exacerbator phenotype. Chest.

[CIT0011] Muñoz-Esquerre M, Diez-Ferrer M, Montón C (2015). Roflumilast added to triple therapy in patients with severe COPD: a real life study. Pulm Pharmacol Ther.

